# Laboratory analogue of a supersonic accretion column in a binary star system

**DOI:** 10.1038/ncomms11899

**Published:** 2016-06-13

**Authors:** J. E. Cross, G. Gregori, J. M. Foster, P. Graham, J. -M. Bonnet-Bidaud, C. Busschaert, N. Charpentier, C. N. Danson, H. W. Doyle, R. P. Drake, J. Fyrth, E. T. Gumbrell, M. Koenig, C. Krauland, C. C. Kuranz, B. Loupias, C. Michaut, M. Mouchet, S. Patankar, J. Skidmore, C. Spindloe, E. R. Tubman, N. Woolsey, R. Yurchak, É. Falize

**Affiliations:** 1 Clarendon Laboratory, University of Oxford, Parks Road, Oxford OX1 3PU, UK; 2 AWE, Aldermaston, Reading, West Berkshire RG7 4PR, UK; 3 Service d‘Astrophysique-Laboratoire AIM, CEA/DSM/Irfu, 91191 Gif-sur-Yvette, France; 4 CEA-DAM-DIF, F-91297 Arpajon, France; 5 First Light Fusion Ltd, Unit 10 Oxford Industrial Park, Mead Road, Yarnton Oxfordshire, OX5 1QU, UK; 6 Department of Atmospheric, Oceanic and Space Sciences, University of Michigan, Ann Arbor, Michigan 48109, USA; 7 LULI-CNRS, Ecole Polytechnique, CEA: Université Paris-Saclay; UPMC Univ Paris 06: Sorbonne Universités-F-91128, Palaiseau Cedex, France; 8 Institute for Academic Initiatives, Osaka University, Suita, Osaka 565-0871, Japan; 9 LUTH, Observatoire de Paris, PSL Research University, CNRS, Université Paris Diderot, Sorbonne Paris Cité, 92190 Meudon, France; 10 Target Fabrication Group, Central Laser Facility, Rutherford Appleton Laboratory, Harwell Science and Innovation Campus, Didcot OX11 0QX, UK; 11 York Plasma Institute, Department of Physics, University of York, Heslington, York YO10 5DQ, UK

## Abstract

Astrophysical flows exhibit rich behaviour resulting from the interplay of different forms of energy—gravitational, thermal, magnetic and radiative. For magnetic cataclysmic variable stars, material from a late, main sequence star is pulled onto a highly magnetized (*B*>10 MG) white dwarf. The magnetic field is sufficiently large to direct the flow as an accretion column onto the poles of the white dwarf, a star subclass known as AM Herculis. A stationary radiative shock is expected to form 100–1,000 km above the surface of the white dwarf, far too small to be resolved with current telescopes. Here we report the results of a laboratory experiment showing the evolution of a reverse shock when both ionization and radiative losses are important. We find that the stand-off position of the shock agrees with radiation hydrodynamic simulations and is consistent, when scaled to AM Herculis star systems, with theoretical predictions.

## Abstract (web summary)

Stationary radiative shocks are expected to form above the surface of highly-magnetized white dwarves in binary systems, but this cannot be resolved by telescopes. Here, the authors report a laboratory experiment showing the evolution of a reverse shock when both ionization and radiative losses are important.

## Graphical abstract

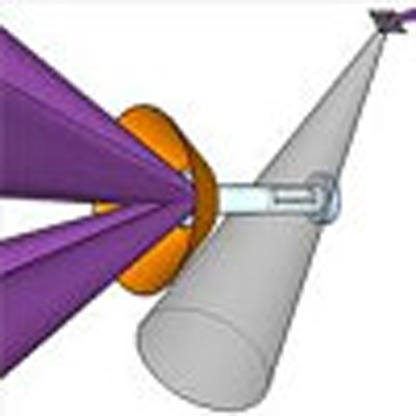

Shocks, waves and jets are important in interstellar and circumstellar regions, and their dynamics can be significantly altered in the presence of radiative losses and magnetic fields^1^,^2^. Radiative shocks and ionization fronts are examples of such phenomena. By acting as an energy sink, radiative cooling, trapping and ionization modify the shock structure and properties away from that expected in an ideal gas. This is important in star formation^3^,^4^ and in supernovae explosions^5^ where a dense shell of material can be formed, with compression much above what would normally be expected from an ideal strong shock.

Laboratory experiments offer an alternative way to study ionizing and radiative shocks, and to probe them in a detailed way that would not be possible in space. High power lasers, such as the Orion Laser Facility, Aldermaston (UK)^6^, can produce plasmas of sufficient density, velocity and temperature that are astrophysically relevant^7^,^8^. This is possible because of the hydrodynamic similarity that can be established between the laboratory and the astrophysical systems, which has been investigated in depth^9^,^10^,^11^,^12^,^13^,^14^,^15^, and, recently, also include the full combination of magnetohydrodynamics, radiation and quantum effects^16^.

One such astrophysical event that shows ionizing and radiative shocks is the white dwarf accretion column in a magnetic cataclysmic variable (MCV) star system—see ref. 17 for a review—where material from a late, main sequence star is pulled off by a highly magnetized white dwarf, a star subclass known as AM Herculis. Instead of the typical accretion disc, the strong magnetic fields (*B*>10 MG) cause the plasma fluid from the secondary star to follow the magnetic field lines of the white dwarf. When the magnetic field strength is ∼10–30 MG, bremsstrahlung emission dominates the cooling process^18^,^19^, and, as the flow travels perpendicular to the field, the magnetic field only acts to contain the plasma. The field directs the flow onto the poles of the white dwarf, where it has an impact and a radiative reverse shock is formed, which travels counter to the incoming flow. Theories relating the properties of the star to the shock height^20^, and thus the accretion mode^21^, have implications for interpreting observational data, such as the ratio of hard to soft X-ray emission^22^. However, the distance of the shock above the white dwarf photosphere is too small for telescopes to resolve.

Here we show a scaled laboratory experiment, building on previously developed platforms^20^,^23^,^24^,^25^,^26^, to investigate such astrophysically relevant radiative shocks. An analogue of the astrophysical system is produced and an experimental regime reached such that radiation is beginning to have important effects, (*id est*, material and radiative energy fluxes are of similar orders of magnitude), which has an impact on the compression across the shock and the shock stand-off distance. Indeed, our measured shock compression ratio is higher than for classical hydrodynamic shocks. We also observe the shock stand-off distance, when scaled to MCVs, is in very good agreement with the expected position of the reverse shock from the white dwarf surface. In future, this platform could be extended to larger laser systems to study hydrodynamics in radiation-dominated environments.

## Results

### X-ray radiographs

**Figure 1:**
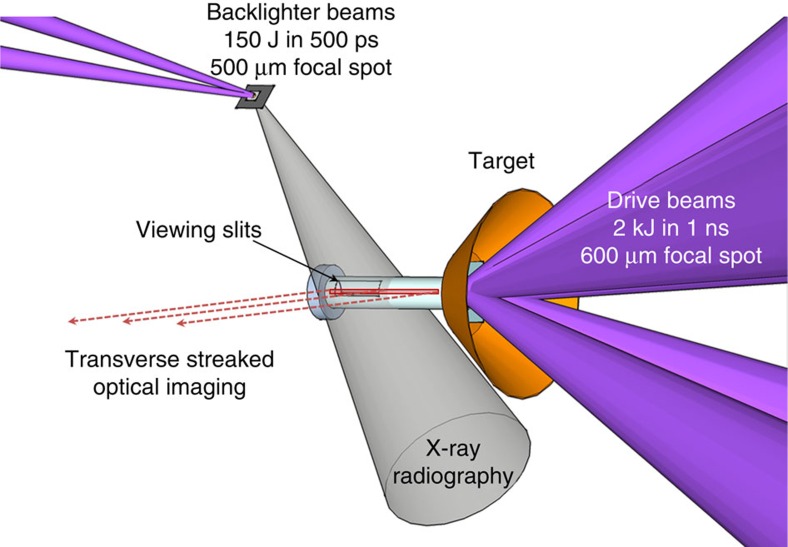
Schematic of the experimental set-up. The laser beams from the Orion facility are arranged in two cones, which we refer to as ‘drive' and ‘backlighter'. The drive cone consisted of five beams, which illuminated the target in a 100° cone angle, with each beam supplying ∼400 J (2 kJ in total) in 1 ns onto a 600-μm spot, which gave an average intensity of 7.0 × 10^14^ W cm^−2^. For the backlighter cone, we used two laser beams of total energy 150 J, in 500 ps onto a 500-μm spot, which gave an average intensity of 1.5 × 10^14^ W cm^−2^. Both sets of beams were delivered at the third harmonic with a wavelength of 351 nm. Viewing slits, along the X-ray diagnostic line of sight, were cut on either side of the tube at the obstacle end.

**Figure 2:**
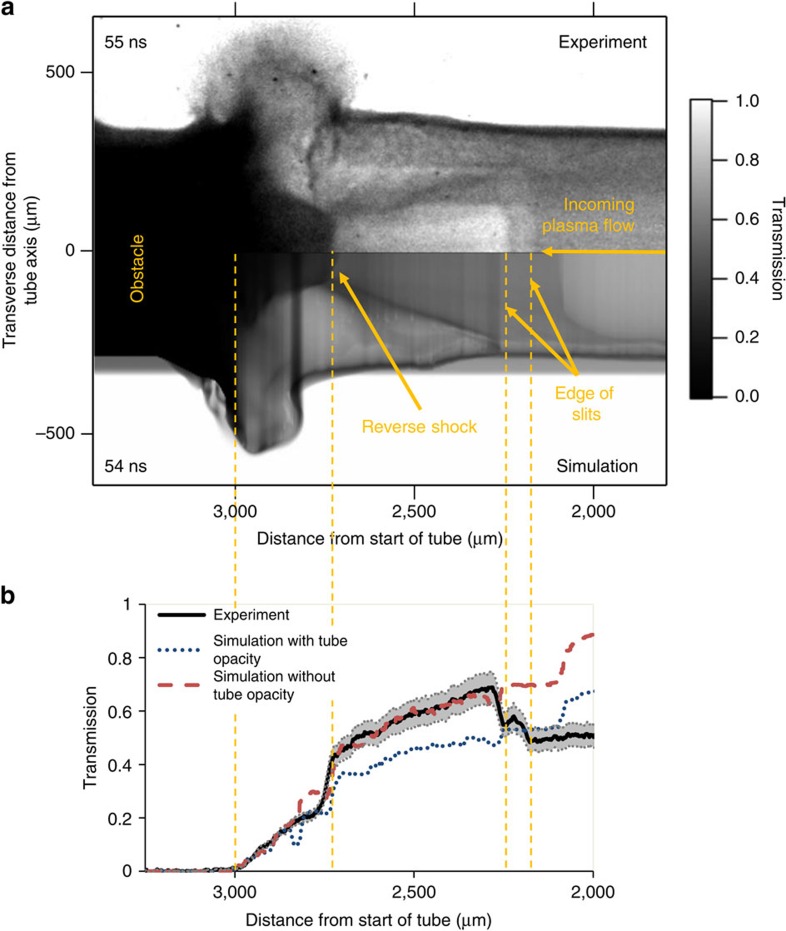
Comparison of experimental and simulated results. A secondary target was used as an X-ray source for backlit pinhole radiography of the reverse shock region (see Fig. 1 and Methods). The plasma flow enters from the right of the figure and impacts onto the obstacle, originally at 3,000 μm, forming a reverse shock that can be seen around 2,750 μm. The top half of (**a**) shows the experimental radiograph, with the bottom half of (**a**) showing a synthetic radiograph from postprocessing a 2D simulation. The target tube had a slit cut into it from ∼2,200 to ∼3,100 μm. The same radiative hydrodynamic simulation was postprocessed to give simulated radiographs with and without the effect of the tube opacity, to account for the effect of the tube being absent at the slit position. A line-out of X-ray transmission along the central axis, for the experimental case and postprocessing of the simulated case with and without the opacity of the tube wall, is plotted in (**b**). The transmission is well matched for the experimental case and simulated radiograph without tube opacity from the position of the reverse shock, ∼2,750 μm, to the edge of the slit, ∼2,200 μm. After this point, the experiment transmission agrees better with the simulated radiograph including the tube opacity.

The experimental setup is illustrated in Fig. 1 (see also Methods). Figure 2a shows a comparison of simulated and experimental X-ray radiographs of the plasma having an impact on the obstacle. A radiation-hydrodynamic numerical simulation was performed using a suite of different codes (see Methods) and postprocessed to generate two different synthetic X-ray radiographs, one including the opacity of all the target components, *id est*, the standard case, and another with the opacity of the plastic tube artificially set to zero to mimic signal seen through the viewing apertures, cut in the experimental target tube. As shown in Fig. 1, the plastic tube in the experiment had slits cut through it, near the obstacle, to improve contrast by allowing X-rays to go through only the plasma and not to be absorbed by the tube wall. The experimental and simulated X-ray radiographs shown in Fig. 2 are quantitatively similar not only in the morphology of the flow structure—where the position of the reverse shock front is nearly identical—but also in the X-ray transmission values, which are well matched. We can see in the experimental image that the slits were not exactly aligned along the X-ray diagnostic direction by the step down in transmission ≲2,250 μm and again at ≲2,200 μm. The transmission values (Fig. 2b) from the synthetic radiograph without the tube wall opacity are consistent with the experimental data near the reverse shock (that is, between 2,300 and 3,000 μm), whereas the simulated transmission with the tube wall included is closer to the data for distances ≲2,300 μm. The jump in the simulated transmission around 2,100 μm, which is not seen in the experiment, results from the ablator plasma (which is more transparent to X-rays) immediately following the pusher plasma. It is also worth noting that the experimental results are reproducible: a later shot at the same time as Fig. 2 also shows the same features and shock stand-off distance (see Supplementary Fig. 1 and Supplementary Discussion: Radiograph comparison).

### Optical self-emission

**Figure 3:**
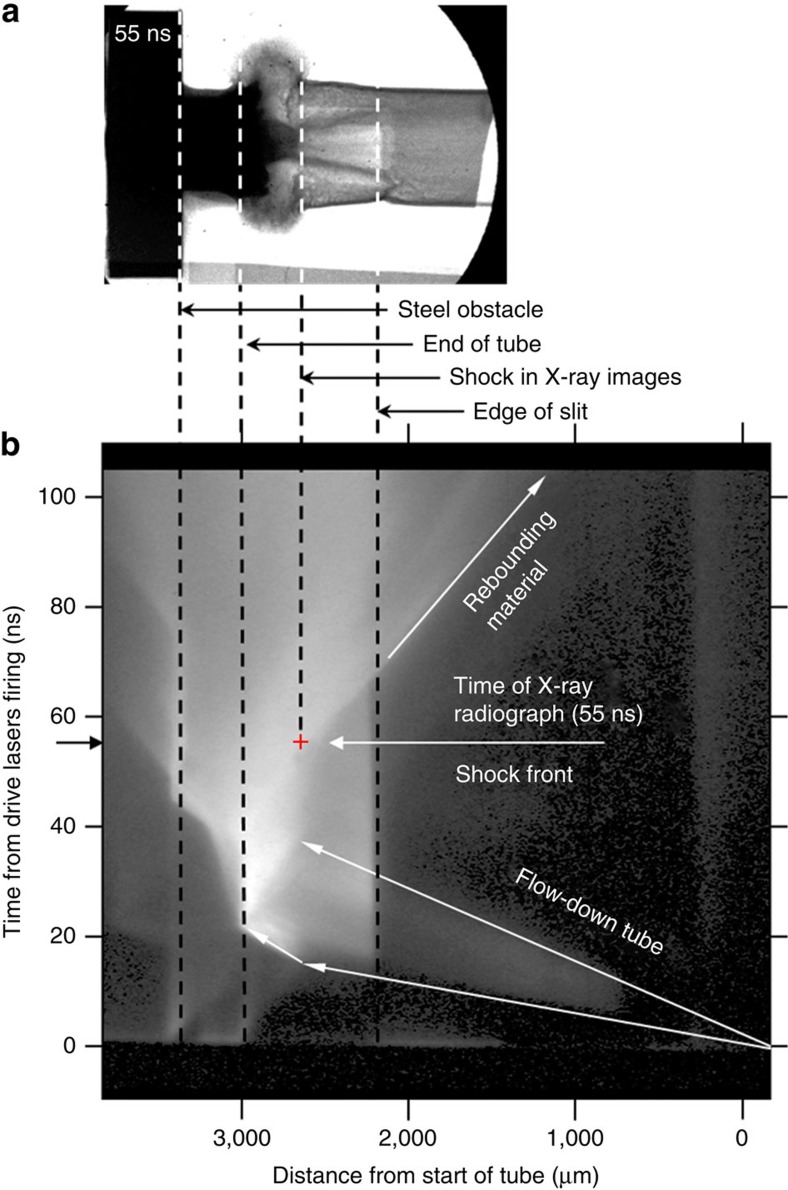
Streaked optical image of the flow and shock evolution. (**a**) An X-ray image taken from Fig. 2 aligned with the streaked optical image in (**b**). Optical diagnostics were fielded in the experiment to record the self-emission from the hot plasma along the entire length of the tube. An imaging system directed the light emitted by the interacting plasma to the streak camera. The streak camera had a spatial field of view of ∼4 mm and thus the entire tube and obstacle were visible, except the region shielded by the copper cone. An imaging line aligned with the central axis of the tube (along the slit cut-out in the tube), in the horizontal plane, was then streaked in time over ∼60, 100 or 250 ns as required. This provided a temporal dependence of flow velocities in plasma. Time runs from bottom to top, distance right to left. The time evolution of the plasma can clearly be seen inside the tube. The dotted lines and overlaid radiograph show positions of parts of the target on the streaked image. There is good agreement in the position of features between the X-ray (**a**) and optical (**b**) diagnostics.

Figure 3 shows an image of the optical self-emission streaked in time. Time *t*=0 ns starts when the drive lasers illuminate the target foils and increases moving up the image. The drive lasers are incident on the foil at 0 μm and distance increases from right to left. The experimental image indicates that the velocity of plasma flowing down the tube is 70±10 km s^−1^ in the laboratory frame, which agrees with the predicted velocity from numerical simulations. As the flow reaches the obstacle, there is an initial build-up of material, which eventually steepens into a shock at *t*∼40 ns. The shock position in the laboratory frame changes very slowly for *t*≲55 ns and subsequently begins to move back up the tube. The brightening in the image for distances ≳2,200 μm is due to increased emission where the tube wall is absent (here the slit in the tube faces the optical diagnostics, unlike Fig. 2 where the windows face the X-ray diagnostics). The position of the shock is marked by a cross and the overlaid X-ray radiograph at *t*=55 ns shows good agreement between the diagnostics. The numerical simulation also predicts a slowly varying shock front stand-off position, albeit at slightly earlier times, between 38 and 50 ns. This is probably due to limitations of a two-dimensional (2D) simulation capturing the full dynamics of the flow.

### Numerical simulations

**Figure 4:**
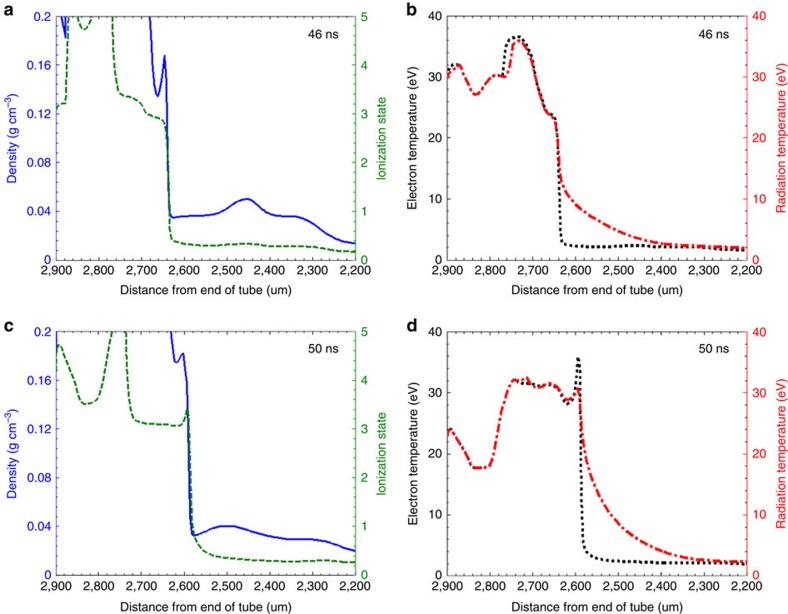
Prediction from numerical simulations. Lineouts, from the central axis of a 2D simulation, of various plasma properties are shown at two different times. Density and ionization state (**a**,**c**) are shown as solid blue and dashed green lines, respectively; electron temperature and radiation temperature (**b**,**d**) are shown as dotted black and dot-dashed red lines, respectively. At the shock position we see an abrupt change in all four of these properties.

The good agreement between the experimental X-ray images and the synthetic ones obtained from postprocessing the 2D simulations, as well as their prediction of the overall structure of flow gives us confidence in the estimates of the microscopic properties of the plasma. The results for the simulations are shown in Fig. 4. In particular, we notice that the predicted reverse shock exhibits a sudden jump in both density and ionization fraction.

Assuming that the gold and plastic materials are uniformly mixed, and that the total opacity is simply the weighted sum of the opacity of each component (see Supplementary Fig. 2 and Supplementary Discussion: Compression calculation and Mixing of gold-plastic layers in pusher foil), the density jump in the reverse shock between the upstream and downstream flows is estimated to be ∼5.6±0.6 at *t*=55 ns (where the error comes from taking backlighter energies in a range from 3 to 4 keV; the energy of the backlighter was inferred to be ∼3.75 keV, using transmission through a step wedge. See Supplementary Fig. 3 and Supplementary Methods: X-ray energy). This agrees well with the density jump calculated in the simulations (∼5.5, as shown in Fig. 4 at 50 ns).

## Discussion

Using standard equations for the conservation of mass and momentum across the shock transition region, ignoring the radiation pressure term (which is negligible under these conditions), and using the thermal pressure *p*_*i*_ (*i*=1 and 2 for the upstream flow and downstream plasma, respectively) we obtain^27^:


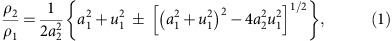


with 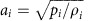 the isothermal speed of sound, *ρ*_*i*_ is the mass density and *u*_1_ the upstream flow velocity.

Equation (1) admits a solution only when *u*_1_≥*u*_R_ or *u*_1_≤*u*_D_, where the rarified and dense velocities, respectively, are defined as 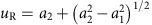 and 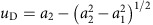. If *u*_1_>*u*_R_, then the flow is supersonic and it results in a compression of the downstream plasma. This requires us to take the positive root in equation (1) and is expected in the reverse shocks occurring in both the MCV star system and in our experiment. The scaling between the two systems is given in Table 1. Values for the incoming flow velocity are taken from our streaked optical data (Fig. 3) and sound speeds are calculated using simulated values for the temperature. The Reynolds number is large in the astrophysical system and the laboratory, indicating that viscous dissipation can be neglected. The thin radiation number—a measure of the incoming material energy flux compared with the radiation flux^16^—is very small in the astrophysical case and by no means large in the laboratory. This implies that radiation losses cannot be ignored and are important in determining the overall evolution of the flow. This is also reflected by the radiation cooling time 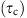 being of the order of the characteristic dynamical time.

Using the simulated flow velocity (*u*_1_∼83 km s^−1^ in the frame of the shock), temperature and ionization fraction (Fig. 4), we estimate, using equation (1), *ρ*_2_/*ρ*_1_≈5.7±0.6, whose value agrees with that inferred from the X-ray radiographs (where the error is calculated by allowing a 10% variation in thermal pressure when calculating the sound speeds). Equation (1) follows from the mass and momentum equations, and is not affected by details relating to ionization and radiation.

The compression ratio, in the strong shock limit (which applies in our experiment), is given by 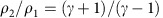, where 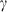 is the ratio of specific heats. Taking the compression value as 5.6, from the experiment, we estimate, from the ideal jump condition, an effective 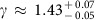.

The adiabatic exponent, taken from the relation of the energy, density and pressure in the ideal case^28^, is given by 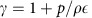, where *ε* is the internal energy. Using values taken from the simulation gives γ=1.4 which agrees with the value of γ taken from the experiment. Ionization of the material at the shock front thus causes a greater densification of the material. The presence of a spike in the simulated electron temperature at the shock front (∼2,590 μm in Fig. 4 at 50 ns) suggests the presence of radiative effects^28^. Following the analysis by ref. 24 in determining whether radiative effects are important, the flow minus the shock velocity must equal or exceed 83 km s^−1^. From the experimental and simulated numbers, we see that we are just in this regime.

In the astrophysical case, the stand-off position of the shock front with respect to the surface of the white dwarf is estimated by^29^,^30^


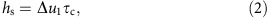


where Δ is a value depending on the dimensionless parameters of the system, *u*_1_ is the incoming flow velocity to the shock front and 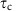 is the cooling time^11^. The form of this equation is valid in a general sense for both optically thin and thick plasmas. The value of delta has little sensitivity to the dynamics, varying between 0.25 and 0.1 according to the dominant radiative process. In the optical streak image in Fig. 3, the reverse shock appears to maintain a steady distance *h*_s_≈200 μm from the obstacle between 40 ns≲*t*≲55 ns. This value is consistent with the prediction of equation (2) and it scales to a stand-off distance of 1,300 km for the MCV system, which is the typical spatial extension as predicted by theory. In the astrophysical case this is mediated by the radiative losses against the incoming mass flux and, hence, radiative effects are very important. However, in the laboratory the radiative and material energy fluxes are of similar magnitude. Owing to the low temperature of the post-shock region in laboratory plasma, a microscopic, scaled model between the laboratory and astrophysics cannot be produced. Indeed, the laboratory plasma does not radiate primarily by bremsstrahlung cooling, but instead by line emission. However, as the radiative processes modify the macroscopic structure of the flow, as also occurs at astrophysical scales, we can use the parametric similarity introduced by Falize *et al.*^10^,^11^ to relate the laboratory plasma to the astrophysical flows. Thus, the relation in equation (2) is applicable in the laboratory and astrophysics, and the results can be related to one another.

Our work provides a laboratory platform to investigate the physics of the accretion column near the white dwarf photosphere, which is not currently accessible by observational techniques. Future experiment at larger lasers such as the National Ignition Facility or Laser MegaJoule could increase the radiative nature of the reverse shock and comprehensively test how radiative losses affect the stand-off distance. This can have important consequences for the explanation of the luminosity curve in variable star systems, as the position of the shock above the white dwarf photosphere determines the ratio of hard and soft X-ray emission^22^, which itself is dependent on the accretion model^21^.

## Methods

### Laser and target

The drive beams were used to produce an expanding plasma jet, whereas the backlighter beams generated an X-ray source that imaged the plasma flow and the reverse shock formation. The main target consisted of a 550-μm inner diameter, 650-μm outer diameter and a 3.5-mm-long polyimide tube, with foils affixed at one end and an obstacle at the other. The laser spot size was deliberately chosen to be larger than the inner diameter of the tube so as to be less susceptible to misalignment and thus preserve a relatively uniform transverse profile of the plasma flowing down the tube. The drive beams illuminated the end of the target capped with two foils: a 25-μm-thick plastic ablator and a 25-μm-thick gold–plastic layered, pusher foil. The pusher was manufactured by depositing alternating layers of CH and Au, with 6-μm-thick CH and 300-nm-thick Au, giving a total Au concentration in the pusher of 39% by mass. A copper cone was placed just behind the ablator and pusher foils to protect the diagnostics from the direct view of the laser spot and from emission from the blow-off plasma. The other end of the tube was sealed with a steel obstacle, which was inserted ∼150 μm into the tube. In some targets, ∼1-mm-long slits were cut into the tube near the steel obstacle, at 45° to the horizontal plane. This allowed for clearer imaging of the optical and X-ray emission from the interacting plasma. See also ref. 31 for additional details on the target.

### X-ray target

This target consisted of a 400-μm diameter chlorinated plastic (parylene-D) foil, which was then affixed onto a 50-μm CH foil, and finally placed onto a large tantalum foil with a 15-μm pinhole. This target was placed 14.1 mm from the steel obstacle and illuminated by the backlighter beams (see Fig. 1). The backlighter beams overfilled the target, with a focal spot of 500 μm, which caused an expansion of the surrounding plastic and the target material as well. This acted to contain the blow-off and the tantalum pinhole ensured a point-like source of X-rays, which backlit the main target and were recorded on an image plate detector placed 229 mm from the main target^32^. This gave a field of view of ∼2 mm and a magnification of ∼15. The energy of the X-rays are expected to be mainly from Cl line emission: *id est*, He-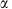 at 2.78 keV, He-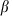 at 3.27 keV, Ly-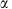 at 2.96 keV and Ly-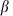 at 3.50 keV (see also Supplementary Fig. 4). Appropriate filters were added in front of the image plate: a 12.5-μm polyvinylidene chloride (PVDC) to cut off lower energies and 5 μm Ti to cut off higher energies, giving an effective window of transmission between 2.78 and 5 keV. A step-wedge filter of 50-,100-and 150-μm-thick plastic was also placed over the image plate detector to calibrate the X-ray emission energy on each shot. See Supplementary Fig. 3 and Supplementary Methods: X-ray energy, for more detail.

### Simulations

In the calculations, the NYM Lagrangian code^33^ was used for the laser-interaction phase, as this allowed fine zoning of the ablator foil, which is essential to resolve light absorption from inverse bremsstrahlung. X-ray emission and absorption were simulated with full multi-group Monte Carlo photonics^34^ using CASSANDRA opacities^35^ and SESAME equations-of-state^36^. After the laser pulse had ended and the coronal plasma had cooled substantially, the simulation was linked to the PETRA code^37^ to continue to late time. This used Eulerian hydrodynamics (run on an orthogonal mesh), which is essential to permit the large shear flows of material along the tube walls. This calculation was restricted to multi-group diffusion for the X-ray exchange but the opacities and equations-of-state were unchanged from NYM. The laser was modelled as a 1-kJ beam normal to the surface and a super-Gaussian spatial intensity profile with 1/e intensity at 300 μm radius. The laser energy was adjusted in order for the predicted flow velocity and the overall flow behaviour to match the experimental case. This gave an effective energy coupling of ∼50%, which is reasonable as the experimental beam angle was not modelled. An electron-conduction flux limiter of 0.05 was used, typical of laser-plasma modelling at this irradiance level. See Supplementary Figs 5 and 6, Supplementary Table 1, Supplementary Discussion: Laser intensity in simulation and Supplementary Methods: Numerical simulations, for more detail on the sensitivity of the simulations.

### Data availability

The data that support the findings of this study are available from the corresponding author upon request.

## Additional information

**How to cite this article:** Cross, J. E. *et al.* Laboratory analogue of a supersonic accretion column in a binary star system. *Nat. Commun.* 7:11899 doi: 10.1038/ncomms11899 (2016).

## Supplementary Material

Supplementary Information Supplementary Figure 1-6, Supplementary Table 1, Supplementary Discussion and Supplementary Methods

## Figures and Tables

**Table 1:** Scaling between the laboratory and the astrophysical system.

**Characteristic quantity**	**Astro**	**Lab**
Length	10^6^ m	1.5 × 10^−4^ m
Incoming flow density	10^−8^ g cm^−3^	0.03 g cm^−3^
Initial flow velocity	1,000 km s^−1^	200 km s^−1^
Incoming flow temperature	0.3 eV	2.2 eV
Post shock temperature	10,000 eV	28 eV
Cooling time 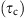	1 s	3.8 × 10^−9^ s
Reynolds number	10^6^	2.2 × 10^5^
Radiation number (Thin)	10^−16^	2.3
Mach number	>10	8
*u*_R_	1,000 km s^−1^	57 km s^−1^
*u*_D_	0.5 km s^−1^	0.55 km s^−1^
Shock height (*h*_s_)	∼1,300 km	200 μm

The scaling relations and the characteristic dimensionless constants are defined as in ref. 16. Values for the magnetic cataclysmic variable star system are taken from ref. 20.
