# Supplementary material for: Laboratory analogue of a supersonic accretion column in a binary star system
Source: Nat Commun. 2016 Jun 13;7:ncomms11899. doi: 10.1038/ncomms11899 (PMC4910020; doi:10.1038/ncomms11899)
Supplement: Supplementary Information — Supplementary Figure 1-6, Supplementary Table 1, Supplementary Discussion and Supplementary Methods [file ncomms11899-s1.pdf]

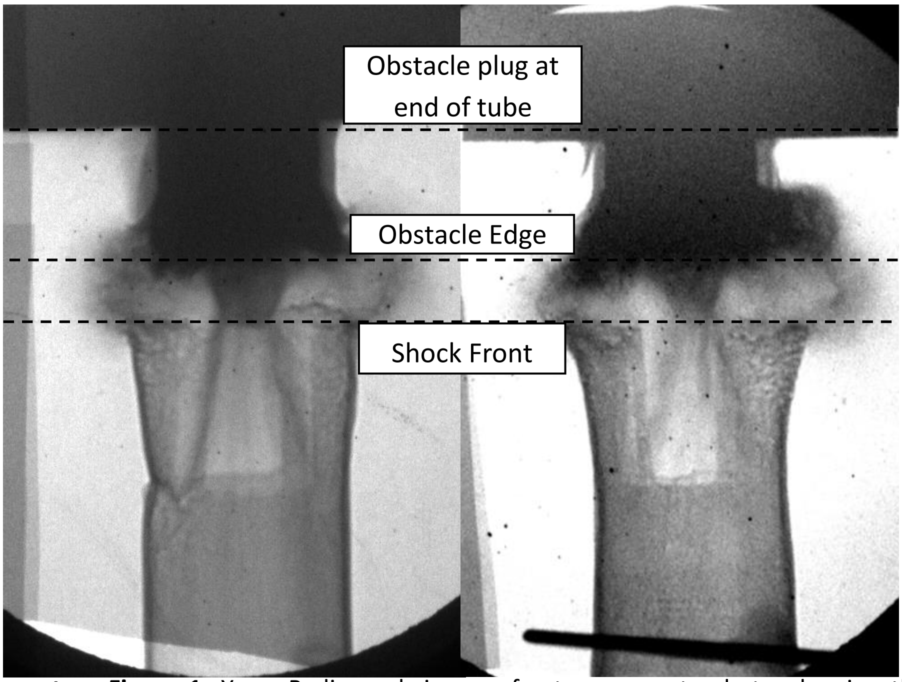


**Supplementary Figure 1**: X-ray Radiograph images for two separate shots, showing the high degree of repeatability through the experiment. The left-hand image is seen in the top panel of figure 2 in the main manuscript. The position of various features is labelled.

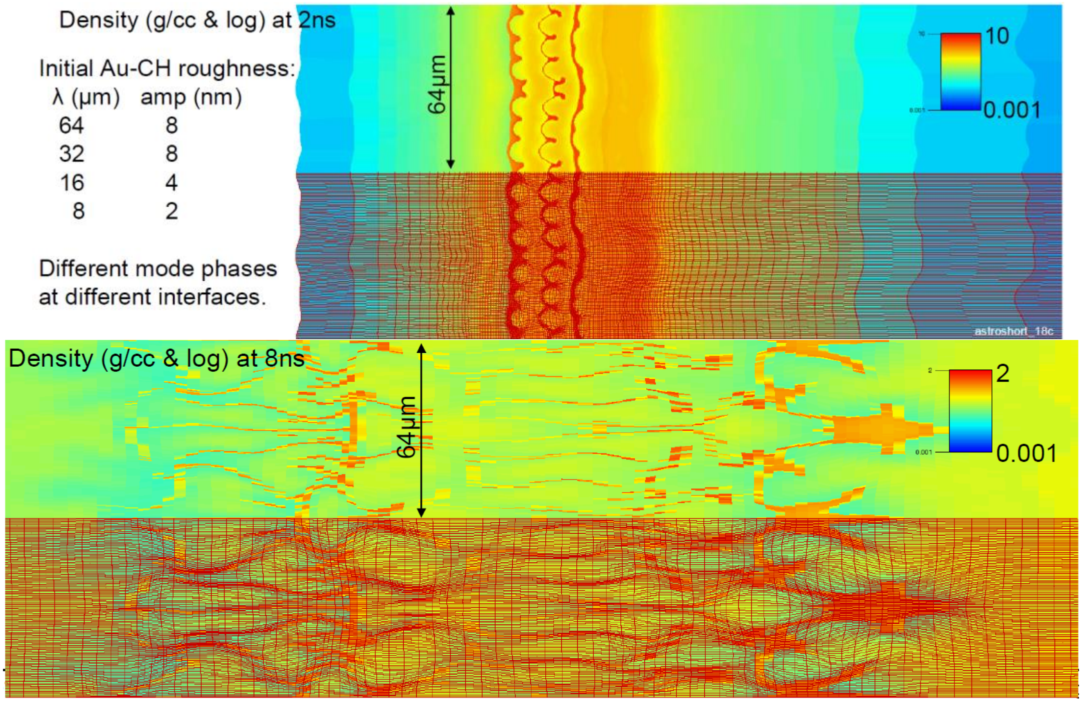


**Supplementary Figure 2**: Simulations showing mixing of gold and plastic layers in the target at early times after the laser is incident. A multi-mode spectrum is imposed on the plastic (CH) – gold (Au) interface to simulate surface roughness, shown in the top left. Denser gold regions, red, and less dense plastic, yellow, can be seen in the top diagram, 2 ns after the laser fired. After 8 ns, bottom diagram, the density is much more uniformly mixed. In each panel, the lower half shows the simulated grid.

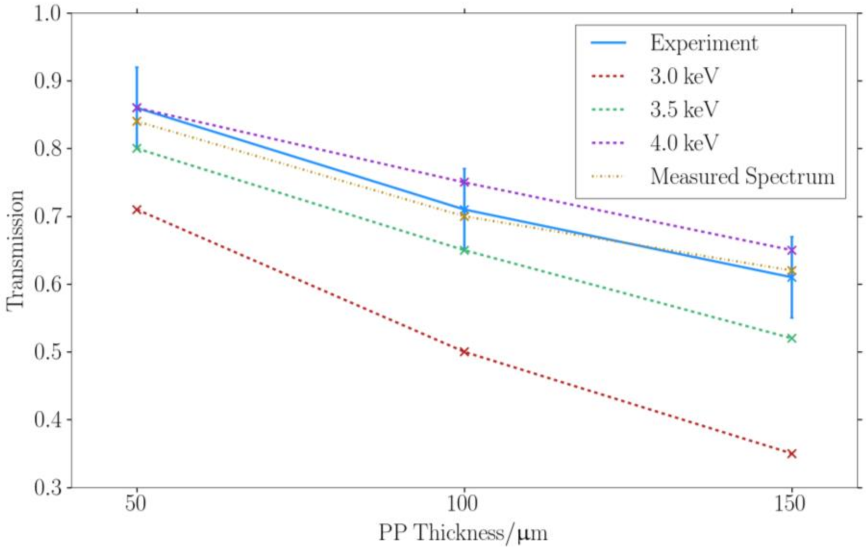


**Supplementary Figure 3:** Showing the expected transmission through the step wedge (PP thickness) for different X-ray energies, compared to the recorded transmission in the experiment. The expected transmission for the measured spectrum (dot-dash in gold) agrees very well with the transmission calculated from the experimental image (solid blue).

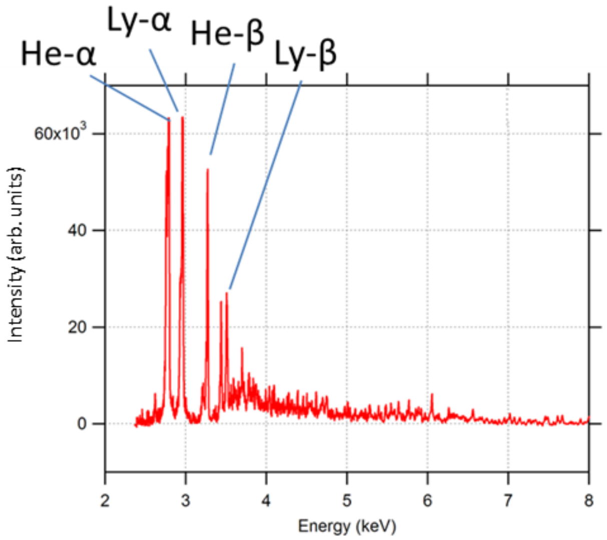


**Supplementary figure 4:** Spectrum of a Chlorine X-ray backlighter. The main contributions to the spectrum from line emission are labelled.

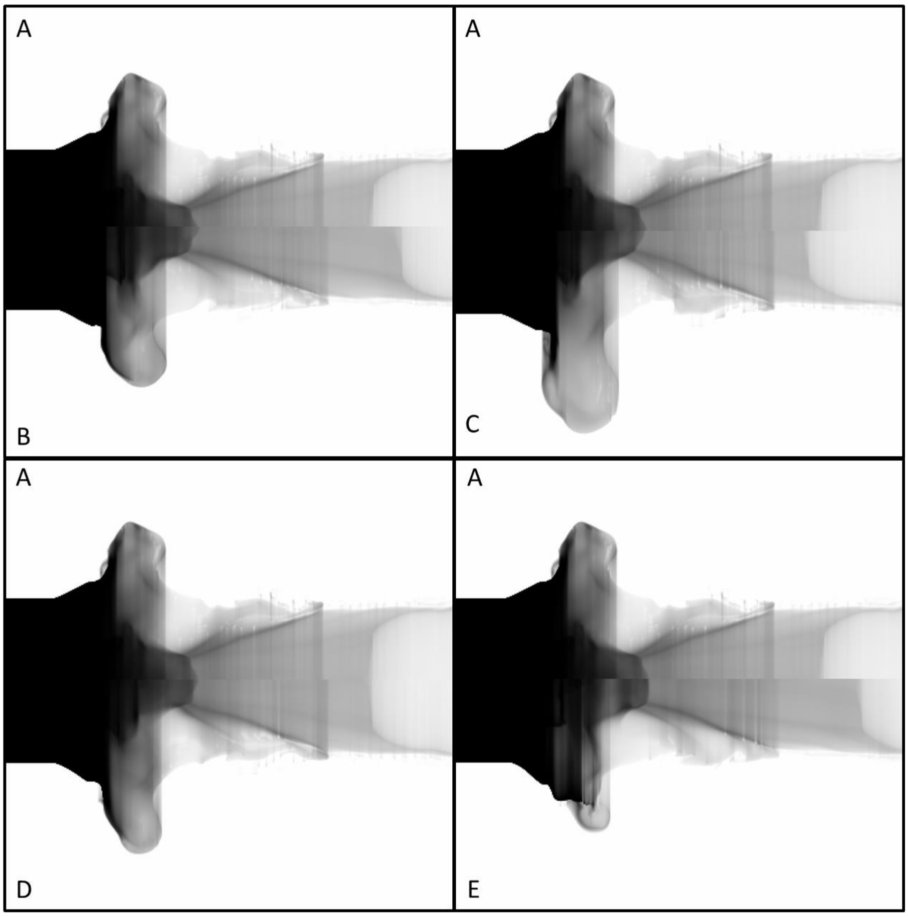


**Supplementary Figure 5**: Comparison between simulated X-ray radiographs. In each panel, the top half image corresponds to run A, the original simulated case. The bottom image of each panel shows the alternative simulations with different EoS and opacities as given in supplementary table 1.

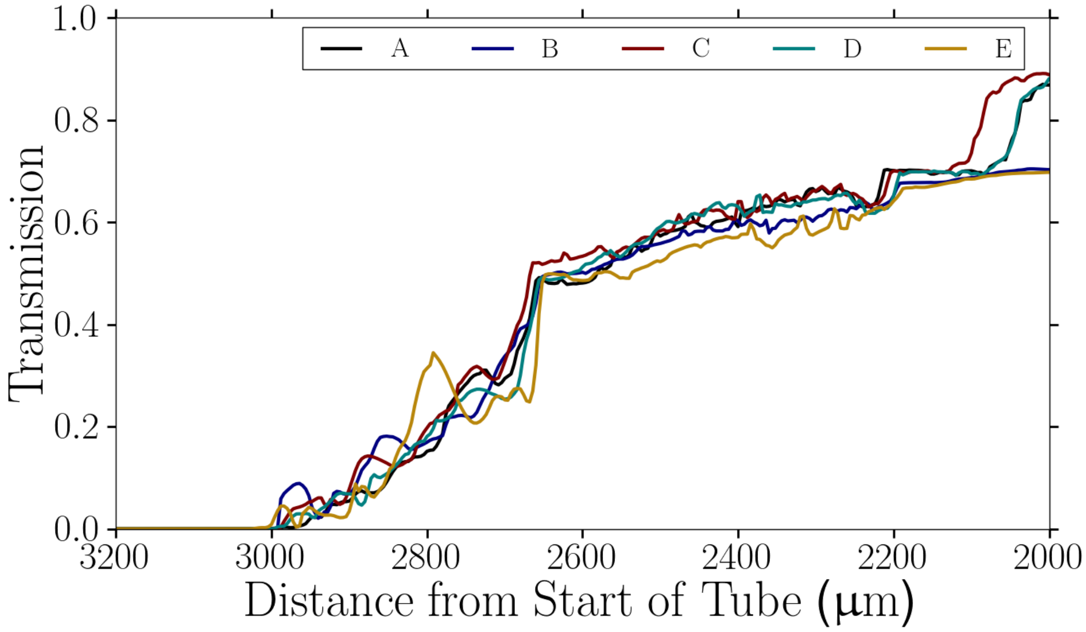


**Supplementary Figure 6**: Predicted x-ray transmission curve through the target for different simulation runs A, B, C, D & E, as given in supplementary table 1. Compare with Figure 2 (bottom panel) in the main manuscript.

# Supplementary Table

| Run | Comment | EoS | Opacity | Time | Colour |
|---|---|---|---|---|---|
| **A** | Original simulation as shown in main text | CHAu (40%) | CHAu (40%) | 54 ns | — |
| **B** | Using PPS (C6H4S) EoS for CH-Au | PPS (C6H4S) | CHAu (40%) | 52 ns | — |
| **C** | Scaling the CH-Au opacities by 20% | CHAu (40%) | 1.2 x CHAu (40%) | 55 ns | — |
| **D** | Using 25% wt Au (instead of 40%) for the opacity | CHAu (40%) | CHAu (25%) | 54 ns | — |
| **E** | Using pure CH EoS for the CH-Au | CH | CHAu (40%) | 51 ns | — |

**Supplementary Table 1:** List of the different numerical simulations performed in this study as seen in supplementary figures 5 and 6 (for cases B-E, the time listed corresponds to the time the shock reaches the same position as in case A). Colours refer to those seen in supplementary figure 6.

# Supplementary Discussion

## Radiograph comparison

Supplementary figure 1 shows a comparison of two different experimental X-ray radiographs taken at the same time delay, 55 ns, after the main drive lasers have fired. In both cases the same gold-plastic layer pusher foil was used as the plasma source. The drive laser intensity was similar in both cases (7.0 x $10^{14}$ W $cm^{-2}$ and 7.4 x $10^{14}$ W $cm^{-2}$ respectively).

The dashed lines show the spatial position of the shock feature. The obstacle edge and shock front are very similar in both cases. The slight difference in the position of the obstacle plug at the end of the tube between the two images is as a result of target to target variation in obstacle orientation, within the allowed tolerances.

## Compression Calculation

Compression was calculated using the following assumptions:

1) The spatial extent of the region of interest was the tube diameter.
2) The opacity was calculated as a weighted sum of the individual component opacities: 0.39 * gold opacity + 0.61 * plastic opacity, and plasma was considered fully mixed (see below).
3) The opacity was considered not to change across the shock front. See supplementary discussion – X-ray opacity.

4) In the region ahead of the shock we have: $\ln\left(\frac{I}{I_0}\right) = -\kappa\rho_u l_u$ where $(I/I_0)$ is the transmission, $\kappa$ is the weighted opacity as defined above, $\rho_u$ is the unshocked density, $l_u$ is the chord length the X-ray travels through, set here to be the tube diameter.
5) In the post-shock region we have: $\ln\left(\frac{I}{I_0}\right) = -(\kappa\rho_u l_{u_s} + \kappa\rho_s l_s)$, where $l_s$ is the spatial extend of the shock front, such that $l_{u_s} + l_s = l_u$, and $\rho_s$ is the shocked density.
6) The equations in 4) and 5) can be solved using the experimental radiograph values in PSL, to give the individual densities, and their ratio.

**Mixing of Gold-Plastic Layers in Pusher Foil**

We have performed simulations of mixing of plastic and gold layers, as a result of Rayleigh-Taylor instability, laser imprint etc. These are shown in supplementary figure 2 and they indicate that significant mixing has already occurred at 8 ns. This suggests that the assumption of uniformity at times around 55 ns is valid. (The effect of roughness between the plastic-gold layer interfaces is imposed by a multi-mode spectrum in the simulation. The simulation in supplementary figure 3 represents a best case for smoothness: it is likely that the actual target had a much rougher interface, which simulations show increase the rate of mixing between the two layers.)

**Laser Intensity in Simulation**

The nominal intensity of the laser (7.0 x $10^{14}$ W $cm^{-2}$), reported in figure 1 caption, does not include losses occurring in the laser beam path and/or due to various laser-plasma instabilities. Those are typically dependent on the angle of incidence and notoriously difficult to model. To estimate such losses, it is easier instead to look at the resultant flow behaviour. For the simulation, an intensity of 3.5 x $10^{14}$ W $cm^{-2}$ was used. If the simulation accurately captures the absorption of the laser, then values of the flow properties obtained from the code, most notably the velocity, should agree with the experimental ones. Indeed, this is the case, as seen under the Optical self-emission of the Results section, thus we have reasonable confidence that the simulation is modelling the laser absorption correctly.

**X-ray Opacity**

As a result of changes in temperature, density and ionisation state, the mean (frequency integrated) opacity will change across the shock front. However, it is the frequency dependent opacity, at the probe X-ray energy, which is of importance when estimating the density. The X-ray energy is much greater than the plasma temperature (3.75 keV X-rays compared to the ~30 eV plasma temperature) and therefore we do not expect the X-ray opacity to vary greatly across the shock front. This is confirmed in the supplementary information where supplementary figure 2 shows that the X-ray transmission does not appreciably change between the different runs

# Supplementary Methods

**Numerical Simulations**

We have run a range of 1-D and 2-D simulations in order to assess the sensitivity of the calculations with different equations-of-state (EoS) and opacities of the materials (see supplementary table 1). In all cases the results were very similar to those shown in the main manuscript (case A in supplementary table 1), including the reverse-shock compression factor.

In 1-D the reverse-shock compression factor varies from 5.2 to 5.9 for the above examples. And in 2-D the flow dynamics evolve very similarly to the original simulation run – shown in figure 2 (top panel) of the main manuscript. This is referred to as case A in supplementary table 1, and for this particular run the reverse shock is predicted to arrive at the position seen in the experimental images about 54 ns after the start of the laser drive. For runs B to E, the shock reaches the same position as the one of run A at very similar times – as shown in supplementary table 1. Supplementary figure 5 gives a clear comparison between the different 2-D simulation runs, with a substantial quantitative agreement for all cases considered here.

In supplementary figure 6 we have also compared the post-processed x-ray transmission through the target (omitting the tube walls) with that reported in figure 2 (bottom panel) in the main manuscript. Run A in supplementary figure 5 corresponds to the dashed line of figure 2 (bottom panel). As we can see, for all runs, the predicted x-ray transmissions are very similar to each other, confirming the weak dependence of our estimates on the details on the EoS and opacity models.

Sensitivities to the laser parameters were not investigated as the simulations are tuned to match the shock propagation and position in the experimental data and so the laser drive is constrained.

## X-ray Energy

The X-ray energy, for each shot, was determined by fitting the measured x-ray signal transmitted through a step wedge placed on the image plate. By this method, it was found that the "effective" X-ray energy is between 3.5 keV and 4.0 keV, and this was the range used in the estimates done in the main paper. This energy range was further confirmed by a measurement of the x-ray spectrum of the backlighter target alone.

To estimate the effective x-ray energy by the step wedge method, layers of 50 µm, 100 µm and 150 µm thick polypropylene were placed on the image plate. The transmission onto the image plate was recorded and compared to the expected transmission for different X-ray energies. This can be seen in supplementary figure 3. Also overlaid is the expected transmission through the step wedge for the X-ray spectrum that was recorded from the backlighter target only. The transmission from the experimental image and the expected transmission from the recorded X-ray spectrum of the backlighter foil are indeed in very good agreement.

The spectrum of the X-ray backlighter alone is given in supplementary figure 4. This shows emission from He-α at 2.78 keV, He-β at 3.27 keV, Ly-α at 2.96 keV and Ly-β at 3.50 keV, as expected, as well as continuum radiation.
